# Assessing reactive astrogliosis in aging and across the Alzheimer's disease spectrum with SMBT‐1

**DOI:** 10.1002/alz70856_103477

**Published:** 2025-12-26

**Authors:** Victor L Villemagne

**Affiliations:** ^1^ University of Pittsburgh, Pittsburgh, PA, USA

## Abstract

**Background:**

Monoamine Oxidase‐B (MAO‐B) is overexpressed in reactive astrocytes around Aβ plaques. We characterized the performance of ^18^F‐SMBT‐1 ‐a MAO‐B PET tracer‐ in aging and across the AD continuum.

**Method:**

We quantified ^18^F‐SMBT‐1 PET regional binding in young (25‐38yo) and elderly cognitively unimpaired (CU) and elderly cognitively impaired (MCI and AD) (age range 61‐88yo). Participants also underwent Aβ and tau PET imaging, 3‐T MRI, neuropsychologic evaluation and fluid biomarker assessment.

**Results:**

^18^F‐SMBT‐1 showed robust entry into the brain and reversible kinetics, with regional tracer binding in Aβ‐ CU following the known regional brain distribution of MAO‐B (R2=0.84). ^18^F‐SMBT‐1 also captured the known MAO‐B increases with age. Parameters derived from full compartmental analysis with arterial input function were highly correlated with a simple 70‐90min tissue ratio using the cerebellar cortex as pseudoreference region. There was a strong association between plasma GFAP and regional ^18^F‐SMBT‐1 PET, and this association appears to be dependent on brain insoluble Aβ load, not soluble plasma Aβ. In several regions in the brain, especially those known for early Aβ deposition, ^18^F‐SMBT‐1 was highly associated with Aβ load, and much less with tau. In a longitudinal cohort examining the effects of cardiovascular risk factors (CVRF) on AD pathology, ^18^F‐SMBT‐1 binding was associated with an early, non‐canonical/Aβ‐independent high tau deposition.^18^F‐SMBT‐1 showed that Aβ+ve AD patients, but most importantly, Aβ+ve CU individuals, had significantly higher regional ^18^F‐SMBT‐1 binding than Aβ‐ve CU participants. These findings suggest that increased ^18^F‐SMBT‐1 binding is detectable at the preclinical stages of Aβ accumulation. Longitudinal follow up of these participants showed not only a different brain regional behavior across the AD continuum, but Aβ‐ve CU participants with high ^18^F‐SMBT‐1 accumulated Aβ while CU Aβ‐ve CU participants with low ^18^F‐SMBT‐1 did not, indicating that high ^18^F‐SMBT‐1 in Aβ‐ve CU is predictive of future Aβ accumulation.

**Conclusion:**

^18^F‐SMBT‐1 is a robust surrogate marker of regional reactive astrogliosis. It is highly correlated with Aβ burden and with plasma GFAP. High ^18^F‐SMBT‐1 precedes and predicts high Aβ suggesting reactive astrogliosis is an interesting target for early therapeutic interventions.

